# Unusual duplication of the insulin-like receptor in the crustacean *Daphnia pulex*

**DOI:** 10.1186/1471-2148-10-305

**Published:** 2010-10-12

**Authors:** Philippe Boucher, Delphine Ditlecadet, Caroline Dubé, France Dufresne

**Affiliations:** 1 Université du Québec à Rimouski, Département de Biologie, Centre d'Études Nordiques, 300 Allée des Ursulines, Rimouski, Québec, G5L 4L8 Canada; 2 Department of Biology; Memorial University of Newfoundland; St. John's, Newfoundland, A1B 3X9 Canada

## Abstract

**Background:**

The insulin signaling pathway (ISP) has a key role in major physiological events like carbohydrate metabolism and growth regulation. The ISP has been well described in vertebrates and in a few invertebrate model organisms but remains largely unexplored in non-model invertebrates. This study is the first detailed genomic study of this pathway in a crustacean species, *Daphnia pulex*.

**Results:**

The *Daphnia pulex *draft genome sequence assembly was scanned for major components of the ISP with a special attention to the insulin-like receptor. Twenty three putative genes are reported. The pathway appears to be generally well conserved as genes found in other invertebrates are present. Major findings include a lower number of insulin-like peptides in *Daphnia *as compared to other invertebrates and the presence of multiple insulin-like receptors (InR), with four genes as opposed to a single one in other invertebrates. Genes encoding for the Dappu_InR are likely the result of three duplication events and bear some unusual features. Dappu_InR-4 has undergone extensive evolutionary divergence and lacks the conserved site of the catalytic domain of the receptor tyrosine kinase. Dappu_InR-1 has a large insert and lacks the transmembranal domain in the β-subunit. This domain is also absent in Dappu_InR-3. Dappu_InR-2 is characterized by the absence of the cystein-rich region. Real-time q-PCR confirmed the expression of all four receptors. EST analyses of cDNA libraries revealed that the four receptors were differently expressed under various conditions.

**Conclusions:**

Duplications of the insulin receptor genes might represent an important evolutionary innovation in *Daphnia *as they are known to exhibit extensive phenotypic plasticity in body size and in the size of defensive structures in response to predation.

## Background

The insulin signaling pathway is evolutionary well conserved among multicellular organisms. In humans and higher vertebrates, signaling through the insulin pathway is critical for the regulation of intracellular and blood glucose levels and has a pivotal importance in metabolic diseases, such as diabetes, and cellular process such as ageing [1-3]. It is also well recognized as a major regulator of growth in both vertebrates and invertebrates and can also trigger diapause and regulate ageing in invertebrates [4-8].

The insulin signaling pathway is initiated by the secretion of insulin like peptides (ILPs), in response to elevated glucose and amino acid levels [9]. Binding of ILP initiates a complex cascade of events, starting with phosphorylation of specific tyrosine residues on the insulin/IGF-like receptors (InR) [10]. Once activated, these receptors phosphorylate a number of docking proteins; the best characterized are the insulin receptor substrate (IRS/Chico) proteins [11]. IRS interact with other intracellular signaling molecules primarily through src homology 2 (SH2) domains leading to the activation of several downstream pathways. These in turn coordinate and regulate vesicle trafficking, protein synthesis, and glucose uptake [12]. The InR and its substrates, therefore, constitute the first critical node in the insulin signaling network and thus define the full set of proteins that are tyrosine phosphorylated upon ILP stimulation.

The InR belongs to an ancient transmembrane receptor tyrosine kinase (RTK) superfamily [13]. Unlike the other members of the RTK family, the InR forms a tetramer of two extracellular alpha (*α*) subunits, linked by disulphide bonds to two beta (*β*) subunits, which pass through the cell membrane and have intracellular tyrosine kinase domains. Both *α *and *β *chains are synthesized from a single mRNA with a variable number of exons [14]. Although there is an exceptional evolutionary conservatism in the function and components of insulin signaling pathway, major changes have occurred between vertebrates and invertebrates in the beginning of the pathway. First, the early duplication events in vertebrates gave rise to specialized receptors, the insulin receptor, the type 1 insulin growth factor receptor (IGF1R) and the insulin receptor-related receptor (IRR) [15]. The primary function of the insulin receptor is to control blood sugar homeostasis while the IGF1R promotes pre and post-natal growth. These receptors show a relatively high specificity with their respective ligand [16]. The IRR is called an orphan receptor as the ligand and its biological function is still unknown.

The insulin signaling pathway has been extensively characterized in the fruitfly *Drosophila melanogaster *and the worm *Caenorhabditis elegans *and partially described for a large variety of invertebrates from sponges to insects [17-21]. Recent studies have revealed that invertebrates possess many more insulin-like peptides than expected, up to 37 in *C. elegans *[22]. Although there are numerous insulin-like peptides in invertebrates, only one insulin/IGF receptor homolog has been described to date in all of them (but two distinct IR homologs have been found in the parasite trematode *Schistosoma mansoni *[23]). In this study, we report for the first time, the presence of four InR homologs in *Daphnia pulex *(Crustacea, Cladocera). Phylogenetic analyses confirmed that the four receptors belong to the insulin receptor family. We discuss the evolutionary significance of the duplications and highlight the significance of these results for the biology of this species.

## Results

**Figure 1:**
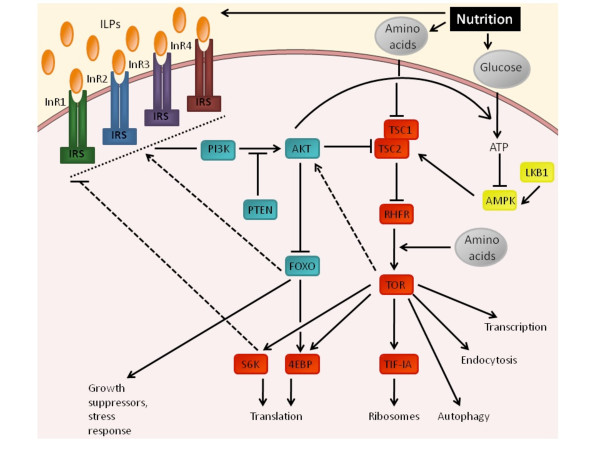
***Daphnia pulex *insulin and TOR signalling pathways, adapted from *Drosophila melanogaster ***[39]. The *D. pulex *insulin/insulin-like growth factors signalling (**IIS**) comprises a group of insulin-like peptides (ILPs), four insulin receptors (InR1, InR2, InR3 and InR4) genes, an insulin receptor substrate (IRS), the type phosphatidylinositol-3-kinase (PI3K p85/p60 and PI3K p110), the lipid phosphatase PTEN, the protein kinase PKB/AKT, the phosphoinositide-dependent protein kinase 1 (PDK-1) and the transcriptional factor FOXO. The TOR- pathway includes a TSC complex (TSC1 and TSC2), a small GTPase RHEB, the target of rapamycin (**TOR**), two S6 kinase (S6K1 and S6K2), the 4E-binding protein (4EBP) and the Pol I transcription factor TIF-1A. The AMPK-pathway involves the activation of AMP-dependent kinase (**AMPK**) by the LKB1 protein kinase.

Analyses of the newly sequenced *D. pulex *genome, the first crustacean draft genome sequence, allowed us to identify key components of the insulin pathway. The *D. pulex *genome includes 25 putative genes belonging to the insulin signaling pathway, which include all major components of this signaling system. *Daphnia pulex *appears to have a relatively low number of insulin-like peptides as compared to other invertebrates but has four putative insulin-like receptors. (Table 1). The rest of the pathway appears similar to what is known from other invertebrates with no appearance of duplication (Figure 1).

**Table 1:** Insulin signaling pathway gene annotation based on the scaffolds_assembly_2 database obtained from Dappu v1.1 draft genome assembly.

Name		Transcript	Location/scaffold:start-endID	Dappu Protein	aa	ORF AT%	intron s
							
Dappu_In R-1	Insulin like Receptor 1	SNAP_00006053	10:1702194-1718363	237995	1865	52,89	14
							
Dappu_In R-2	Insulin like receptor 2	NCBI_GNO_110800001	1108:3248-11233	346810	1358	53,03	15
							
Dappu_In R-3	Insulin like receptor 3	SNAP_00036543	644:5525-14464	268485	1502	52,34	20
							
Dappu_In R-4	Insulin like receptor 4	SNAP_00005849	10:937486-943080	237791	1313	54,72	19
							
Dappu_Ilp-1	Insulin like peptide 1	estExt_fgenesh1_pg.C_470008	47:57736-59749	226060	180	55,88	3
							
Dappu_Ilp-2	Insulin like peptide 2	PASA_GEN_10400015	104:374758-377811	300602	216	58,67	4
							
Dappu_Ilp-3	Insulin like peptide 3	PASA_GEN_1800032	18:435862-436685	302531	152	54,56	3
							
Dappu_Ilp-4	Insulin like peptide 4	leftNCBI_GNO_1800139	18:538680-539524	316719	149	55,66	3
							
Dappu_InRS	Insulin receptor substrate	e_gw1.29.222.1	29:390579-391733	52188	202	53,14	2
							
Dappu_PTEN	Lipid Phosphatase	e_gw1.20.196.1	20:1303190-1304533	49762	280	57,02	5
							
Dappu_PI3-K p85/p60	PhosphatidylinosItol 3-kinase	fgenesh1_pg.C_scaffold_88000090	88:408648-410341	111818	395	42,93	3
							
Dappu_PI3-K p110	Phosphatidylinositol 3-kinase	fgenesh1_pg.C_scaffold_273000007	273:41146-4488	117886	1025	51,59	6
							
Dappu_PKB/AKT	Protein kinase B	leftfgenesh1_pg.C_scaffold_8000380	8:2219257-2222866	299642	532	56,14	7
							
Dappu_PDK-1	Phosphoinositide-dependent protein kinase 1	estExt_fgenesh1_pm.C_740012	74:575211-577498	299641	512	60,58	8
							
Dappu_FOXO	Forkhead transcription factor FOXO	fgenesh1_pg.C_scaffold_62000021	62:204351-207767	109005	608	39,63	3
							
Dappu_TSC1	Tumor suppressor 1	NCBI_GNO_0400016	4:131873-137181	311244	1179	46,55	8
							
Dappu_TSC2	Tumor suppressor 2	leftestExt_fgenesh1_pg.C_1310026	left131:269796-275730	228923	1640	56,49	12
							
Dappu_RHEB	Ras homolog enriched in brain	estExt_Genewise1Plus.C_380048	38:377244-378643	198166	185	63,35	4
							
Dappu_TOR	Target of rapamycin	leftPASA_GEN_11600021	116:299425-310107	347004	2517	56,58	40
							
							
Dappu_S6K1	S6 kinase 1	gw1.9.278.1	9:436444-438032	23503	386	51,9	6
							
Dappu_S6K2	S6 kinase 2	estExt_fgenesh1_pg.C_330055	33:380993-386134	225339	730	59,47	13
							
Dappu_4EBP	4E-binding protein	JGI_CAOC3950	20:1348160-1349320	290539	131	61,14	2
							
Dappu_TIF-IA	Pol I transcripEon factor	estExt_fgenesh1_pg.C_170250	17:970425-973185	223699	546	59,79	6

### Phylogenetic analyses of the receptors

Phylogenetic relationships of Dappu_InR1-R4 with various invertebrate and vertebrate InR were investigated. Tyrosine kinase (TK) domain of diverse RTK and InR were aligned using MUSCLE 3.7 and the alignments were refined with Gblocks 0.91b which eliminates poorly aligned positions and divergent regions in order to focus on unambiguously aligned residues. The RTK and InR alignments used in the phylogenetic analyses included 187 amino acids, which represent 63% of the original 295 positions. Overall, there were 30 conserved positions and 122 parsimony informative sites.

**Figure 2:**
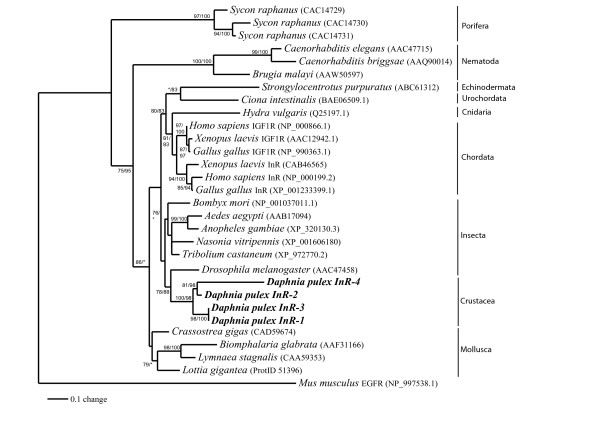
**Phylogenetic tree for insulin receptors tyrosine kinase constructed with maximum likelihood and Bayesian inference methods under the WAG+G model of evolution**. Numbers represent percent bootstrap values (maximum likelihood/Bayesian inference); unlabeled branches indicate a value less than 75. GenBank or Ensembl accession numbers are in brackets.

The ML and Bayesian analyses of the InR using the RTKs of the mouse EGFR as outgroup recovered the phylogenetic tree (ML: - logL = 4403.76) shown in figure 2. The trees were generally reliable, consisting of five major clusters namely porifera, nematodes, molluscs, deuterostomes, and arthropods. The main difference between the phylogenies produced by maximum likelihood and bayesian were branch lengths and the position of *Ciona intestinalis *that branched with *Drosophila *under maximum likelihood but not under Bayesian analyses. The phylogenetic analyses confirmed the monophyly of the four *Daphnia *insulin's receptors suggesting an origin through duplication events. The fourth InR was found to have undergone extensive evolutionary divergence as its branch was much longer than the other InR. The *D. pulex *InR were found to be closely related to those of the other arthropods. The *D. melanogaster *InR showed a surprising grouping with the *D. pulex *InR although the bootstrap support for this phylogenetic relationship was moderate. The phylogeny produced with the complete insulin sequence confirmed the monophyly of the four *D. pulex *InR and the longer branch of *Dappu_InR4 *but placed *D.melanogaster *with the other insect taxa.

### Comparative analyses of the insulin receptors

The length of the open reading frame of the *Daphnia pulex *insulin receptors ranged from 1313 and 1865 amino acids. The A+T composition was very similar among the four receptors. The exon-intron structure of each gene presents a relatively complex and variable structure and the receptors 1 to 4 were respectively composed of 26, 15, 20 and 19 introns (Table 1). Two assembly gaps were found, one of 6 kb in Dappu_InR1 located after exon 13 and another one of 1 kb in Dappu_InR2. Dappu_InR3 and Dappu_InR4 did not show gaps (Additional Figure 1).

**Figure 3:**
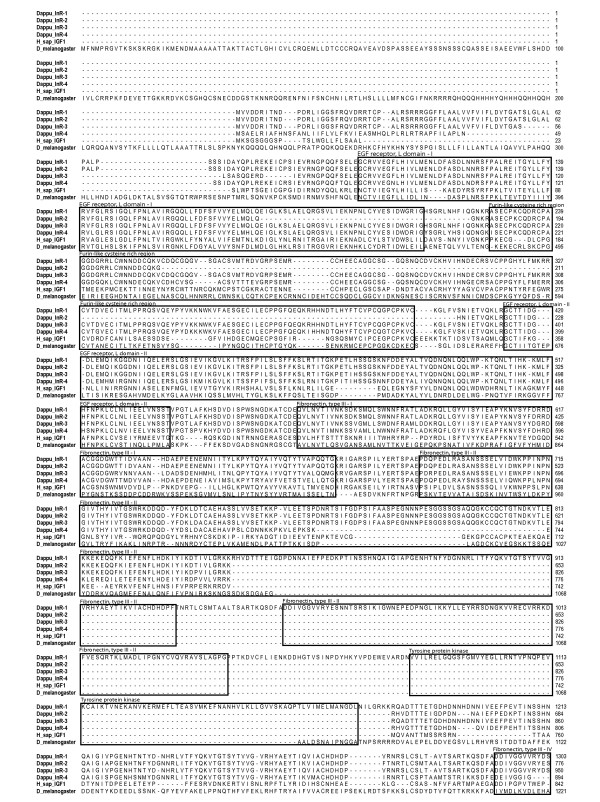
**Amino acid alignment of the four Dappu_InR with *Drosophila *and *Homo sapiens *sequences**.

The insulin receptor is a modular protein containing several specific functional domains. The structural analysis of the *D. pulex *insulin receptors revealed the presence of nearly all of the typical domains found in well characterised insulin receptors such as human or *Drosophila *(Figure 3). Surprisingly, the fourth receptor lacked the conserved site of the catalytic domain of the receptor tyrosine kinase.

The insulin receptor is transmembranal and formed by two a and two b subunits linked in a disulfide b-a-a-b configuration. Dappu_InR1, Dappu_InR2, Dappu_InR3, and Dappu_InR4 possessed a 20 a.a. transmembrane domain in the beginning of the gene almost in the same region as the one identified in *Drosophila *The insulin receptor 2 had a second transmembranal domain of 20 a.a. located upstream into the gene just before the tyrosine protein kinase domain. The structure of the extracellular region of the insulin receptor is predicted to be composed of six distinct structural domains as follows: two homologous domains L1 and L2 flanking a cysteine-rich domain CR followed by three fibronectin type III repeats (Fn0, Fn1, and Fn2). All four analysed receptors included two L domains but the Dappu_InR-2 lacked the cysteine-rich domain CR. Consequently, in this receptor, the two L domains are separated by only one a.a. In the extracellular region, we also identified fibronectin type III domains. Dappu_InR1, Dappu_InR2, Dappu_InR3, and Dapppu_InR4 contained three highly similar fibronectin regions as in *Homo sapiens *and *Drosophila *(region I-II-IV in Figure 3). Dappu_InR1 included an additional fibronectin domain that we identified as an insert. The insertion (333 a.a. in length) was located immediately after a predicted basic cleavage site (KRR847) and had a tyrosine protein kinase domain that itself included a protein kinase ATP binding conserved site.

**Figure 4:**
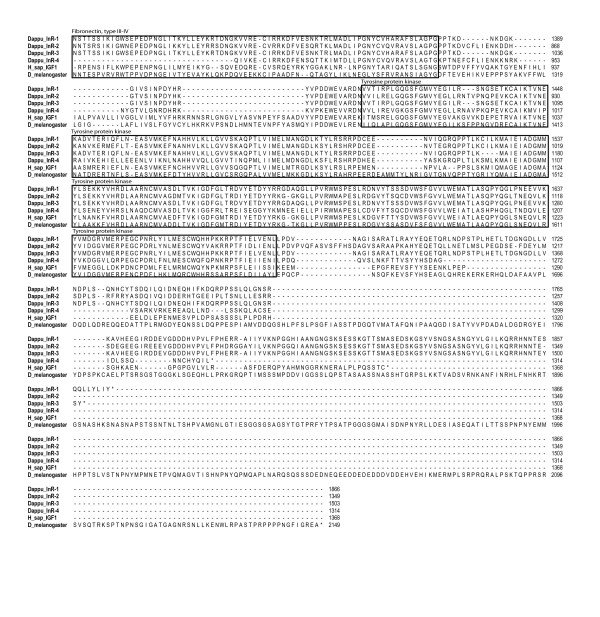
**Amino acid alignment of the four Dappu_InR with *Drosophila *and *Homo sapiens *sequences (continued)**.

The intracellular region of the insulin receptor *i.e*. the cytoplasmic tyrosine kinase activity is responsible for ligand-induced signal transduction to metabolic and mitogenic responses. Compared to the extracellular ligand binding determinant, this portion of the insulin receptor was much more conserved among the four *Daphnia *insulin receptors but also when compared to the other taxa (Figure 4). Dappu_InR1, Dappu_InR2, and Dappu_InR3 exhibited a protein tyrosine kinase catalytic domain signature which included ATP binding site, sequence required for ATP stabilization, motif implicated in phosphotransfer, Mg^2+ ^binding site, consensus "PVRWMAPE" and two juxtaposed autophosphorylation sites characteristic of InR domain (for details on conserved peptide motifs that define the 11 protein kinase subdomains see [24]).

Overall, the analyses of the gene arrangements of the four InR of *D. pulex *revealed that the extracellular parts were more variable than the intracellular components. Dapppu_InR-3 appeared to be the more typical or conserved of the four as Dappu_InR-2 lacked the CR region and Dappu_InR-1 had a long insert including an additional FN type 3 domain as well as an additional protein kinase domain. Dappu_InR4 lacked the catalytic site of the tyrosine kinase.

The dN:dS ratios did not show significant differences in all species tested and in the four *Daphnia *receptors and were mostly lower than one in all pairwise comparisons, thus congruent with purifying selection.

## Expression analyses

Investigations of aligned EST sequences from condition specific cDNA libraries on the Draft genome sequence scaffolds revealed that all four InR were expressed under various conditions. (Table 2). RT-qPCR confirmed the expression of all four receptors. Single products were found for each receptor and efficiencies rates were close to 95%.

**Table 2:** Qualitative expression of four *Daphnia pulex *insulin receptors as revealed by cDNA sequencing of condition specific libraries.

	Life stage	Type of library	Number of clones sequenced	Dappu_InR1	Dappu_InR2	Dappu_InR3	Dappu_InR4
Standard male	Male	Normalised	4224	0	1	0	1
Standard female	Female	Normalised	3456	1	0	0	1
Starvation	Juveniles	Standard	2304	0	1	2	0
Calcium starvation	Mixed	Normalised	3849	0	0	0	1
Low mixed metals	Mixed	Normalised	3456	1	0	0	1
High mixed metals	Mixed	Normalised	3840	0	0	1	0
Low nickel	Mixed	Normalised	4224	1	0	1	2
Titanium	Mixed	Normalised	4224	1	0	1	1
Fullerene	Mixed	Normalised	4224	1	1	0	1
Acid stress	Mixed	Normalised	3840	1	0	1	0
Microcystis	Mixed	Normalised	3072	2	0	1	1

## Discussion

In this study, we provide the first detailed genomic study of the insulin signaling pathway in a crustacean species. As expected, all the components of the insulin signaling pathway were present, confirming the conservation of this pathway in metazoans. Few insulin-like peptides were found in *Daphnia *in contrast to other invertebrates. For example, *D. melanogaster *and *C. elegans *possess 7 and 37 ILPs respectively [22]. We report here the presence of four putative InR in the *D. pulex *genome, a unique case in arthropods. A single InR gene has been described in a wide variety of invertebrates including freshwater mollusks, nematodes and insects. A duplication event of the insulin receptor has also been reported in the helminth *Schistosoma masoni *but the structural and functional particularities of one of these receptors (SmIR-1) argued for its involvement in the parasitic lifestyle of this organism [23]. In vertebrates, two duplication events have led to the presence of three InR paralogs (InR, IGF1R and IRR) [15]. The presence of three or more paralogs in vertebrates but only one gene in invertebrates is a common pattern in many protein families. Our results contrast with this trend. The *D. pulex *genome is recognized as one of the most gene rich metazoan genomes, surpassing the number of overall duplicated genes in the genomes of other well characterized species. Duplication of the InR is thus consistent with large numbers of duplicates for other common gene families in *D. pulex *http://www.biomedcentral.com/series/Daphnia. For example, 75 cytochrome P450 http://www.biomedcentral.com/1471-2164/10/169, 64 ABC proteins http://www.biomedcentral.com/1471-2164/10/170 and 11 hemoglobin genes have already been documented in this species http://www.biomedcentral.com/1472-6793/9/7.

The phylogeny of the *D. pulex *InR is consistent with three possible duplication events with a first duplication of the InR gene that could have led to the ancestor of Dappu_InR-1 and Dappu_InR-3 and to the ancestor of Dappu_InR-2 and Dappu_InR-4, which were both subsequently generated in a second round of duplication. Assembly gaps in two of these InR genes will need to be resolved for a complete understanding, but our analyses of the non-gap regions support the reported biological differences.

Following duplication event, three possible fates are possible for duplicated genes [25]. Briefly, (1) duplicated genes are subject to mutation that can destroy incipient function; (2) during the relaxed selection period following the duplication one copy can acquire a beneficial mutation resulting in new function (neofunctionalization); and (3) the original functions of the single-copy gene may be partitioned between the duplicates (subfunctionalization). In our results, Dappu_InR-4 may have acquired new function based on three lines of evidence: 1) the modification of the TK motif involved in phosphotransfer, 2) the longer branch seen on the phylogeny suggesting an unusual rapid evolution rate of Dappu_InR-4 as compared to other InR, and 3) results from cDNA sequencing showing that this receptor was sampled in libraries created under various conditions. Expression studies will need to be performed to determine if the original function has been partitioned among the four duplicates (subfunctionalization) or if Dappu_InR4 has acquired new function by neofunctionalization. The three other InR appear functional even if their ectodomains revealed some intriguing structural features. In the well described InR of vertebrates, the β-subunit includes a single α-helical hydrophobic transmembrane domain [26]. Transmembrane domain interactions are thought to be involved in negative cooperativity of InR [27]. Among the four InR, only Dappu_InR-2 possesses this transmembranal domain on the β-subunit. However, a transmembranal domain has been identified in all four InR near the N-terminal of the α-subunit. To our knowledge, this is the first report of a second transmembranal domain located in the α-subunit of the InR. This particularity is apparently not restricted to *D. pulex *as we found this domain in insects (e.g. *Tribolium castaneum*, *Nasonia vitripennis *and *Drosophila melanogaster*) and vertebrates (e.g. *Mus musculus*) (data not shown).

Another important difference observed among InR is the absence of the furin-like cystein-rich domain in Dappu_InR-2. The cysteine residues involved in the formation of disulfur bonds of this domain maintain the quaternary structure of the extracellular portion of the receptor [26]. These cysteines are highly conserved in all paralogs across vertebrates. A similar pattern is found in *Drosophila *[28] where the receptor assembles into a quaternary structure but only 2 out of the 6 cysteines described in human InR are conserved.

Experiments with chimeric InR have confirmed that the cystein-rich domain constitutes a part of the insulin/IGF1 binding specifity. In the nematode *C. elegans*, the cystein-rich region is much shorter and contains only two conserved cysteines [29]. Actually, it is not known if the absence of this cystein-rich domain compromises or modifies the activity of the receptor. We observed the presence of a large insert of 333 amino acids in Dappu_InR-1 that included a fourth fibronectin type-3 domain and a TK domain. As this insert contains additional motifs characteristic of InR, it remains possible that this sequence bears a specific function. As there is already evidence from EST that these four InR are expressed, further studies are needed to confirm if they are expressed in different tissues and/or life stages *i.e*. if they have different functions. As *Daphnia *are known to exhibit extensive phenotypic plasticity in body size and in the size of defensive structures in response to predation [30,31], duplications of the insulin receptor genes might represent an important evolutionary innovation in this group.

## Conclusion

The insulin signaling pathway is of pivotal importance in metabolic diseases, regulator of growth and ageing and can also trigger diapause. This pathway has been well described in vertebrate and in few invertebrate model organisms but remains largely unexplored in non-model invertebrates. Our study showed the presence of four InR which are likely the result of three duplication events. All four receptors were found to be expressed. One of these receptors (Dappu_InR-4) has likely changed its function but does not appear to be under positive selection. The three other receptors present some unusual structural features such as the presence of a large insert in Dappu_InR-1, the absence of the transmembranal domain in the β-subunit in the Dappu_InR-1 and Dappu_InR-3, and the absence of the cystein-rich region in Dappu_InR-2. Although these InR have some unusual features, the *Daphnia pulex *insulin signaling pathway presents some characteristics (few ILPs and more than one InR) that are more similar to mammalian ISP which makes it an ideal candidate as a model organism for the study of this pathway. Future studies will examine if other daphniids also possess four InR and what are the evolutionary consequences of these duplications in *Daphnia pulex*.

## Methods

### Sequence retrieval

Molecular databases at the National Center for Biotechnology Information http://www.ncbi.nlm.nih.gov and ENSEMBL http://www.ensembl.org/index.html were screened for vertebrate and invertebrate InR using any combination of words related to insulin receptors, e.g. insulin receptor, IGF receptor, insulin, etc. Blast searches were then performed using several well described vertebrate and invertebrate InR sequences as the queries. Other sequence databases that are publicly accessible were also screened to retrieve unannotated sequences. The following websites were used: Joint Genome Institute (JGI) Genome Portal http://www.jgi.doe.gov/Daphnia/, the Wellcome Trust Sanger Institute http://www.sanger.ac.uk, the Institute for Genome Research http://www.tigr.org/tdb/, Washington University Genome Sequencing Centre http://www.genome.wustl.edu, H-invitational database http://h-invitational.jp, Baylor College of Medicine http://www.hgsc.bcm.tmc.edu, Dictybase http://dictybase.org, *Ciona intestinalis*genome http://genome.jgi-psf.org/ciona/. Protein sequences that were of full length or apparently of full length (presence of initial methionine) were included preferentially over partial sequences, although the later were included for some organisms.

### Annotation of insulin signaling pathway in D. pulex

We searched the *Daphnia pulex *genome http://genome.jgi-psf.org/Dappu1/Dappu1.home.html for candidate InR genes using a combination of key-word queries and the tblastn program. This database included a number of gene prediction sets as well as a combined prediction data set. Identification of putative InR gene orthologs was completed by multiple protein sequence alignments followed by phylogenetic analysis. In addition to the InR genes, we also annotated major components of the insulin signaling pathway (Table 1) using the same methods as described above. We described all component members (determined based on sequence homology) and annotated them using an abbreviation of genus and species name followed by the most common designation (for example, Dappu_InR-1 correspond to the first putative InR in *Daphnia pulex*). Numbering of sequences does not imply homology with those of other organisms but rather chronology of discovery or identification. We suggest that all proteins described in this study, which have not been functionally characterized, be considered as *putative*, until confirmed by appropriate functional assays.

### Sequence alignment and phylogenetic analysis

For phylogenetic analyses, alignments included additional InR sequences delimited by the well conserved tyrosine kinase (TK) subdomain. This portion of the gene was selected to include taxa in which the InR was partially sequenced (e.g. *Sycon raphanus*) and allowed the assessment of the genetic relationships of a greater number of taxa from various groups than with the complete insulin sequence. A total of 30 TK domain sequences were included in the phylogenetic analysis. Amino acid sequences were aligned using MUSCLE 3.7 [32] and the alignment was refined with Gblocks 0.91b [33] which eliminates poorly aligned positions and divergent regions in order to focus on unambiguously aligned residues. The alignment of the ingroup taxa included 187 unambiguously aligned residues with no gap and used for the construction of phylogenetic trees by two different analytical approaches: Maximum likelihood (ML) and Bayesian inference (BI). We used PROTEST version 1.2.6 to select the substitution model that best fit the empirical data set [34]. Then, the ML and Bayesian analyses were performed using the fixed rate amino acid replacement model Whelan and Goldman (WAG) + G (α = 0.82). PHYML version 2.4.4 [35] was used to find the ML tree. The robustness of the inferred tree was assessed using bootstrapping (500 pseudoreplicates) as implemented in PHYML. Bayesian analysis was also performed using MrBAYES software, version 3.1[36]. Runs of 1,000,000 generations were executed, with a sampling frequency of 10, a burn-in parameter of 25,000. Stability of the likelihood scores was assessed in preliminary trials before setting the burn-in parameter. To confirm that the results converged to the same topology, we repeated the analysis three times. Bayesian posterior probabilities were calculated using a Markov chain Monte Carlo sampling approach implemented in MrBAYES v3.1. For both phylogenetic analyses, we used *Mus musculus *epidermal growth factor receptor (EGFR) as outgroup. We also performed the phylogenetic analyses on the complete insulin sequence (but on a restricted number of taxa) to verify if the genetic relationships would be similar to those of the TK domain.

Positive selection was tested using Creevey-McInerney [37] analysis with the Crann program. This analysis compares the number of S and NS substitutions by creating a neighbor-joining tree based on dN values (NS substitutions per NS site) and then determining whether mutations are variable (occur more than once in the tree) or invariable (occur in only one branch of the tree). Four values are compared to those predicted by neutral theory: S variable (SV), S invariable (SI), NS variable (NSV), and NS invariable (NSI) mutations. If the NS value is significantly greater than its S counterpart, then positive selection is detected as nondirectional (NSI>SI) or directional (NSV>SV).

### Daphnia pulex InR domains

The different domains and subdomains were compared to consensus domains using BLAST http://www.ncbi.nlm.nih.gov/BLAST/Blast.cgi, SMART http://smart.embl-heidelberg.de, ScanProsite http://www.expasy.org/tools/scanprosite/ and MotifScan http://myhits.isb-sib.ch/cgi-bin/motif_scan. A set of sequences from human (IGF-1R) and *D. melanogaster *InR were aligned together with those of Dappu InR-1, Dappu InR-2, Dappu InR-3 and Dappu InR-4 using clustalW program (MEGA version 4.0 [38] with the default parameters.

### Expression analyses

#### CDNA libraries

Qualitative expression of the four *Daphnia pulex *insulin receptors was obtained by cDNA sequencing of condition specific libraries. Data were extracted from wFleaBase and Gbrowse http://wfleabase.org/genepage/daphnia/JGI_V11_314199, http://wfleabase.org/genepage/daphnia/JGI_V11_270048, http://wfleabase.org/genepage/daphnia/JGI_V11_268485, http://wfleabase.org/genepage/daphnia/JGI_V11_237791).

Detailed description of cDNA libraries can be found at https://dgc.cgb.indiana.edu/display/daphnia/cDNA+sequencing+project.

#### Real-time qPCR analyses

RT-qPCR was used to confirm the expression of the four receptors. Total RNA was extracted from 10 pooled adult *Daphnia *with no eggs in the brood pouch from a single *D. pulex *clone using a Qiagen RNA extraction kit. RT was then performed on 2 αg of total RNA in 20 αL of 1 × M-MLV buffer (50 mmol·L-1 Tris-HCl, pH 8.3; 50 mmol·L-1 KCl; 3 mmol·L-1 MgCl2; 5 mmol·L-1 DTT) containing 0.5 mmol·L-1 dNTPs mix, 2 μmol·L-1 oligo(dT)23 primer, 200 units M-MLV RT, and 20 units of RNase inhibitor. Primers specific for each receptor were designed based on unique exons (except for Dappu_InR4) from predicted cDNA sequences from FleaBase. RT-qPCR was performed in the myIQ BIORAD. Each reaction contained 2αl of cDNA template, 10 αl of Quantifast SYBRgreen PCR master mix (Qiagen) and 0.5 αM of primers (DpInR1F: 5' CAAACACGTCATCCACAAC3', DpInR1R: 5'GCCGCTTCATAAACTCAAGTAAT3', DpInR2F: 5'GCCGCTTCATAAACTCAAGTAAT3', DpInR2R: 5' GCAATTTGACCGCCAGGATT 3', DpInR3F 5' GAGGGTCAACAATGTAGCTGCTAAC3', DpInR3R: 5' CAAAATTGGCTACGGGCACCTCA3', DpInR4F: 5' AAAGAATGCATTCGCCGGAAGGAC 3', DpInR4R: 5' TTTCCTGTTCCGGCCAATGAAACCGCTCGAA3'). PCR was carried out using the following conditions: 5 min at 95°C, 50 cycles consisting of 20 s at 95°C followed by 30 s at 60°C (InR1), 67°C (InR2,3), and 72°C (InR4). Dissociation curves were performed to test for the presence of primers-dimers.

## Authors' contributions

The data were collected and analyzed by PB, DD, CD, and FD. The manuscript was drafted by PB and FD with contributions by DD. PB, DD, and FD participated in the project design. PB and FD have contributed equally to the manuscript. All authors have read and approved the final manuscript. The authors declare that they have no competing interests.

## Supplementary Material

Additional file 1 **Additional figure 1**. Alignment of the four *Daphnia pulex *insulin's receptors showing exons and gap assembly. Click here for file

## References

[B1] Saltiel AR Kahn CR Insulin signaling and the regulation of glucose and lipid metabolism Nature 2001 414 6865 799 806 10.1038/414799a 11742412

[B2] Holzenberger M Dupont J Ducos B Leneuve P Geloen A Even PC Cervera P Le Bouc Y IGF-1 receptor regulates lifespan and resistance to oxidative stress in mice Nature 2003 421 6919 182 187 10.1038/nature01298 12483226

[B3] Rodriguez S Gaunt T Day I Molecular genetics of human growth hormone, insulin-like growth factors and their pathways in common disease Hum Genet 2007 122 1 1 21 10.1007/s00439-007-0378-3 17534663

[B4] Brogiolo W Stocker H Ikeya T Rintelen F Fernandez R Hafen E An evolutionarily conserved function of the Drosophila insulin receptor and insulin-like peptides in growth control Current Biology 2001 11 4 213 221 10.1016/S0960-9822(01)00068-9 11250149

[B5] Tatar M Bartke A Antebi A The Endocrine Regulation of Aging by Insulin-like Signals Science 2003 299 5611 1346 1351 10.1126/science.1081447 12610294

[B6] Antebi A Genetics of Aging in Caenorhabditis elegans PLoS Genetics 2007 3 9 1565 1571 10.1371/journal.pgen.0030129 17907808 PMC1994694

[B7] Partridge L Bruning JC Forkhead transcription factors and ageing Oncogene 2008 27 16 2351 2363 10.1038/onc.2008.28 18391977

[B8] Sim C Denlinger DL Insulin signaling and FOXO regulate the overwintering diapause of the mosquito Culex pipiens Proc Natl Acad Sc USA 2008 105 18 6777 6781 10.1073/pnas.0802067105 PMC2373331 18448677

[B9] Chan SJ Steiner DF Insulin Through the Ages: Phylogeny of a Growth Promoting and Metabolic Regulatory Hormone Amer Zool 2000 40 2 213 222 10.1668/0003-1569(2000)040[0213:ITTAPO]2.0.CO;2

[B10] Ullrich A Schlessinger J Signal transduction by receptors with tyrosine kinase activity Cell 1990 61 2 203 212 10.1016/0092-8674(90)90801-K 2158859

[B11] White MF The IRS-signalling system: A network of docking proteins that mediate insulin action Mol Cell Biochem 1998 182 1 3 11 10.1023/A:1006806722619 9609109

[B12] Britton JS Lockwood WK Li L Cohen SM Edgar BA Drosophila's Insulin/PI3-Kinase Pathway Coordinates Cellular Metabolism with Nutritional Conditions Dev Cell 2002 2 2 239 249 10.1016/S1534-5807(02)00117-X 11832249

[B13] Hubbard SR Till JH Protein tyrosine kinase structure and function Annu Rev Biochem 2000 69 1 373 398 10.1146/annurev.biochem.69.1.373 10966463

[B14] De Meyts P Whittaker J Structural biology of insulin and IGF1 receptors: Implications for drug design Nature Rev Drug Discovery 2002 1 10 769 783 10.1038/nrd917 12360255

[B15] Hernandez-Sanchez C Mansilla A de Pablo F Zardoya R Evolution of the Insulin Receptor Family and Receptor Isoform Expression in Vertebrates Mol Biol Evol 2008 25 6 1043 1053 10.1093/molbev/msn036 18310661

[B16] Nakae J Kido Y Accili D Distinct and Overlapping Functions of Insulin and IGF-I Receptors Endocr Rev 2001 22 6 818 835 10.1210/er.22.6.818 11739335

[B17] Chistyakova OV Signaling pathway of insulin and insulin-like growth factor 1 (IGF-1) as a potential regulator of lifespan J Evol Biochem Physiol 2008 44 1 1 11 18411507

[B18] Wu Q Brown MR Signaling and function of insulin-like peptides in insects Annu Rev Entomol 2006 51 1 24 10.1146/annurev.ento.51.110104.151011 16332201

[B19] Kurz CL Tan MW Regulation of aging and innate immunity in C-elegans Aging Cell 2004 3 4 185 193 10.1111/j.1474-9728.2004.00108.x 15268752

[B20] Oldham S Hafen E Insulin/IGF and target of rapamycin signaling: a TOR de force in growth control Trends Cell Biol 2003 13 2 79 85 10.1016/S0962-8924(02)00042-9 12559758

[B21] Garofalo RS Genetic analysis of insulin signaling in Drosophila Trends In Endocrinol Metabol 2002 13 4 156 162 10.1016/S1043-2760(01)00548-3 11943559

[B22] Leevers SJ Growth control: Invertebrate insulin surprises! Current Biology 2001 11 6 R209 R212 10.1016/S0960-9822(01)00107-5 11301264

[B23] Khayath N Vicogne J Ahier A Younes AB Konrad C Trolet J Viscogliosi E Brehm K Dissous C Diversification of the insulin receptor family in the helminth parasite Schistosoma mansoni FEBS J 2007 274 3 659 676 10.1111/j.1742-4658.2006.05610.x 17181541

[B24] Hanks SK Hunter T Protein kinases 6. The eukaryotic protein kinase superfamily: kinase (catalytic) domain structure and classification FASEB J 1995 9 8 576 596 7768349

[B25] Lynch M The origins of genome architecture 2007 Sunderland, Mass: Sinauer Associates

[B26] De Meyts P Insulin and its receptor: structure, function and evolution Bioessays 2004 26 12 1351 1362 10.1002/bies.20151 15551269

[B27] Yip CC Ottensmeyer P Three-dimensional Structural Interactions of Insulin and Its Receptor J Biol Chem 2003 278 30 27329 27332 10.1074/jbc.R300021200 12764141

[B28] Fernandez R Tabarini D Azpiazu N Frasch M Schlessinger J The Drosophila insulin receptor homolog: a gene essential for embryonic development encodes two receptor isoforms with different signaling potential EMBO J 1995 14 14 3373 3384 7628438 10.1002/j.1460-2075.1995.tb07343.x PMC394404

[B29] Dlakic M A new family of putative insulin receptor-like proteins in C. elegans Curr Biol 2002 12 5 R155 157 10.1016/S0960-9822(02)00729-7 11882301

[B30] Black AR Dodson SI Demographic costs of Chaoborus-induced phenotypic plasticity in Daphnia pulex Oecologia 1990 83 1 117 122 10.1007/BF00324642 28313251

[B31] Cousyn C Meester LD Colbourne JK Brendonck L Verschuren D Volckaert F Rapid, Local Adaptation of Zooplankton Behavior to Changes in Predation Pressure in the Absence of Neutral Genetic Changes Proc Natl Acad Sc USA 2001 98 11 6256 6260 10.1073/pnas.111606798 PMC33455 11353872

[B32] Edgar RC MUSCLE: multiple sequence alignment with high accuracy and high throughput Nucleic Acids Res 2004 32 5 1792 1797 10.1093/nar/gkh340 15034147 PMC390337

[B33] Talavera G Castresana J Improvement of Phylogenies after Removing Divergent and Ambiguously Aligned Blocks from Protein Sequence Alignments Syst Biol 2007 56 4 564 577 10.1080/10635150701472164 17654362

[B34] Abascal F Zardoya R Posada D ProtTest: selection of best-fit models of protein evolution Bioinformatics 2005 21 9 2104 2105 10.1093/bioinformatics/bti263 15647292

[B35] Guindon S Gascuel O A simple, fast, and accurate algorithm to estimate large phylogenies by maximum likelihood Syst Biol 2003 52 5 696 704 10.1080/10635150390235520 14530136

[B36] Ronquist F Huelsenbeck JP MrBayes 3: Bayesian phylogenetic inference under mixed models Bioinformatics 2003 19 12 1572 1574 10.1093/bioinformatics/btg180 12912839

[B37] Creevey CJ McInerney JO An algorithm for detecting directional and non-directiona lpositive selection, neutrality and negative selection in protein coding DNA sequences Gene 2002 300 43 51 10.1016/S0378-1119(02)01039-9 12468084

[B38] Tamura K Dudley J Nei M Kumar MEGA4: Molecular Evolutionary Genetics Analysis (MEGA) software version 4.0 Mol Biol Evol 2007 24 1596 1599 10.1093/molbev/msm092 17488738

[B39] Edgar BA How flies get their size: genetics meet physiology Nat Rev Genet 2006 7 12 907 916 10.1038/nrg1989 17139322

