# Supplementary figures and images for: Unusual duplication of the insulin-like receptor in the crustacean Daphnia pulex
Source: BMC Evol Biol. 2010 Oct 12;10:305. doi: 10.1186/1471-2148-10-305 (PMC2978223; doi:10.1186/1471-2148-10-305)

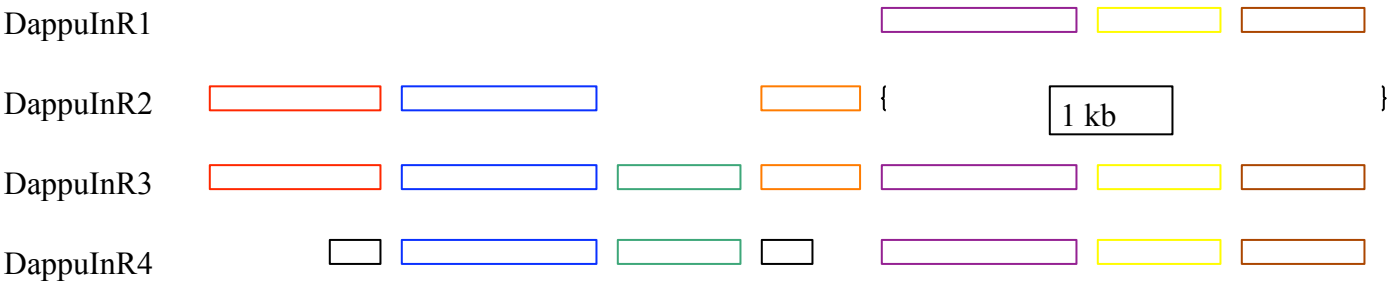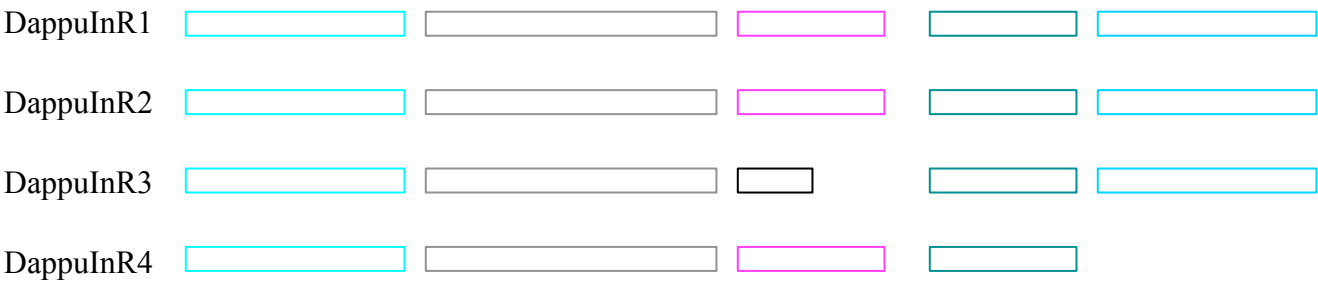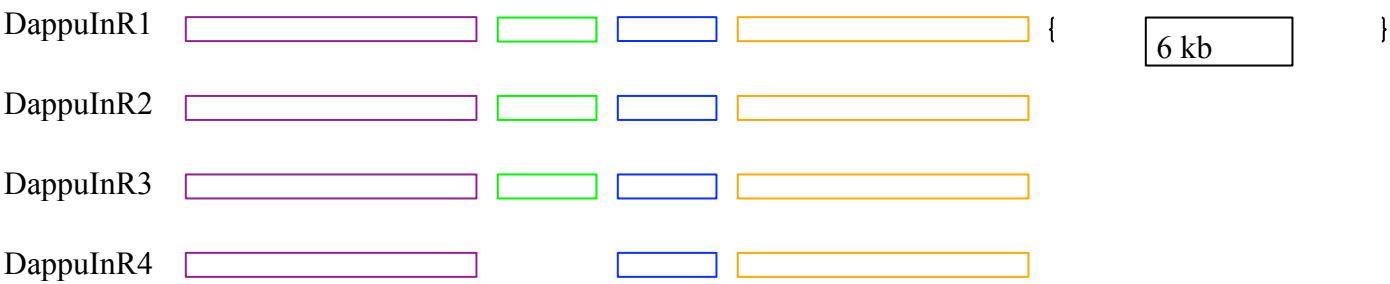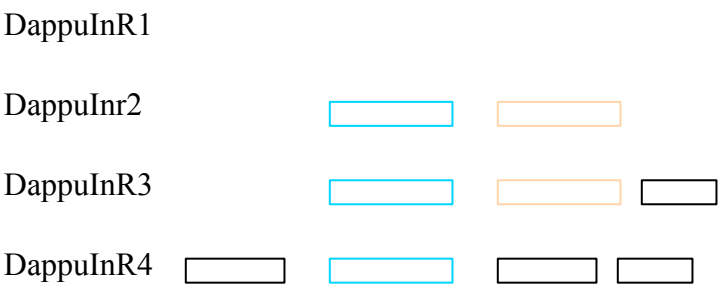

Supplement: Additional file 1 — Additional figure 1. Alignment of the four Daphnia pulex insulin's receptors showing exons and gap assembly. [file 1471-2148-10-305-S1.PDF]
